# Editorial: VIA Character Strengths: Theory, Research and Practice

**DOI:** 10.3389/fpsyg.2021.653941

**Published:** 2021-04-08

**Authors:** Hadassah Littman-Ovadia, Philippe Dubreuil, Maria Christina Meyers, Pavel Freidlin

**Affiliations:** ^1^Department of Behavioral Sciences and Psychology, Ariel University, Ariel, Israel; ^2^Department of Human Resources Management, Université du Québec á Trois-Rivières, Trois-Rivières, QC, Canada; ^3^Department of Human Resource Studies, Tilburg University, Tilburg, Netherlands

**Keywords:** character strengths, VIA inventory of strengths (VIA-IS), VIA classification, virtues, positive psychology

Since the introduction of positive psychology (Seligman and Csikszentmihalyi, 2000), the study of Character Strengths (CS) has been at the forefront of research on human well-being and optimal functioning. Originally developed to provide the field with a foundation for research on what enables and promotes good character and the good life (Peterson and Seligman, 2004), the CS and virtue classification is now considered one of the main building blocks of positive psychology. This classification stems from the early efforts of a group of 55 scientists who undertook the task of systematically reviewing existing psychological, philosophical, and theological literature to identify, classify, and measure universally valued positive traits (Peterson and Seligman, 2004; Dahlsgaard et al., 2005). More specifically, this effort resulted in the identification of 24 CS that serve as “the psychological ingredients—processes or mechanisms—that define the virtues” (Peterson and Seligman, 2004, p. 13). CS can be measured through a variety of assessments (McGrath, 2019), the most popular being the VIA^1^ Inventory of Strengths (VIA-IS), which has been administered to 13,000,000 people worldwide allowing for the ongoing exploration of CS makeup and structure. While originally CS were conceptually categorized under six broad virtues (Table 1), the ongoing administration of the VIA-IS has allowed researchers to empirically examine and update the virtue categories and their ingredients in terms of CS.

**Table 1:** CS and virtues classification.

**Virtues**	**CS**
Wisdom and knowledge	1. Creativity 2. Curiosity 3. Judgment 4. Love of learning 5. Perspective
Courage	6. Bravery 7. Perseverance 8. Honesty 9. Zest
Humanity	10. Love 11. Kindness 12. Social intelligence
Justice	13. Teamwork 14. Fairness 15. Leadership
Temperance	16. Forgiveness 17. Humility 18. Prudence 18. Self-regulation
Transcendence	19. Appreciation of beauty and excellence 20. Gratitude 21. Hope 22. Humor 23. Spirituality

The study of CS has influenced scholarly work across the numerous sub-domains of positive psychology. In the domain of *positive health and wellness*, scholars have explored the relationship between diverse CS profiles and health and well-being, as well as between strengths interventions and well-being (Ghielen et al., 2018; Ruch et al., 2020). Specifically, benefits have been documented in valued outcomes such as general and domain-specific well-being, personal resources, personal growth, performance, and optimal functioning (for reviews, see: Niemiec, 2013; Ghielen et al., 2018; Miglianico et al., 2019; Lavy, 2020; Yan et al., 2020). In the domain of *positive work- and organizational psychology*, scholars have conducted dedicated work on how employees use strengths (Miglianico et al., 2019), employee strengths profiles (Gander et al., 2012), and strengths-based career counseling (Littman-Ovadia et al., 2014). In the domain of *positive clinical psychology*, scholars have reframed psychopathology and clinical symptoms in terms of strengths over-or underuse (Freidlin et al., 2017; Hall-Simmonds and McGrath, 2019). Finally, in the domain of *positive educational psychology*, scholars have investigated strengths-based school counseling (Park and Peterson, 2008) and strengths interventions for children and adolescents (Proctor et al., 2011; Quinlan et al., 2018), among others. It is therefore not surprising that more and more practitioners are applying strength approaches in clinical, counseling, organizational, or educational settings, while others set out to examine and implement CS in novel domains.

Given the time that has passed and the large and varied body of research that has accumulated, we feel that research on strengths has become substantial enough so as to examine its achievements to date, to pause momentarily and evaluate the avenues that have been proposed but left unexplored or understudied, as well as to suggest completely novel directions. As such, the current collection includes 14 articles and illustrates a snapshot of the latest innovative work in CS theory, research and practice.

## Past, Current and Future Perspectives on Character Strengths

The Research Topic (RT) opens with Mayerson's overview of the history of the VIA initiative on character science. Mayerson summarizes research findings on CS to date to offer an integrative model of the role of CS in individual, collective, and species success. He describes “The CS response” as our ability to successfully respond to and navigate various and dynamic life circumstances with the aid of our CS. Because the CS response shows great promise to help our generation, and generations to come, to live good and successful lives, Mayerson argues that there is an urgent need to allocate greater financial and other resources to CS science.

An additional article by Niemiec and Pearce provides a point-in-time examination of crucial CS concepts, definitions and practices. Specifically, they delineate appropriate terminology to distinguish between CS, as well as within CS, in research and practice. Finally, they provide several soaring, emerging and ripe-with-potential CS practices that have been explored to various degrees in research, encouraging cooperation between researchers and practitioners.

Three articles examine the makeup and co-occurrence of the components of good character, each from a different and unique perspective. Ruch et al. provide an account of the co-occurrence of virtues and strengths, measuring the relationships and consistency between CS and their respective virtues, as well as how they are used as ingredients of “good character.” Giuliani et al. provide an additional perspective on the relationship of CS and virtues by introducing a novel “layperson's excellent enactment of highest strengths” paradigm. Specifically, this paradigm demonstrates that describing CS through excellent enactments results in revealing the original six-virtue organization presented by Peterson and Seligman (2004). McGrath and Brown review the VIA Classification of CS and Virtues as an agent to advance the psychological science of virtue, beyond its classic role in the study of positive functioning. In particular, the authors evaluate the available evidence for a three-dimensional cardinal virtue model, including moral, self-regulatory and intellectual domains, to illustrate the evolutionary value of those three domains and provide thoughts on the nature of practical wisdom.

## Wellness and CS Across Life Domains

The current collection expands on the already rich literature on the positive effects of CS in various life domains, further establishing the extensive role of CS in positive functioning. Martínez-Martí et al. empirically examine the associations between CS, subjective well-being, and mental health over the course of a month, during the recent COVID-19 outbreak. The longitudinal design of this study demonstrated a causal relationship in which CS positively affect a variety of outcomes in times of adversity.

Further evidence is presented on vocational CS research. Gander et al. present a novel approach that examines both individual and vocational CS profiles, thereby introducing the person-environment fit paradigm into CS and workplace research, offering additional insight into CS and the effects they have on life and job satisfaction. A supplementary perspective is provided by Gander et al., looking at associations between CS and team roles, and how they affect both individual and team level work outcomes (e.g., performance). They also consider team composition, as rated by the individual CS profiles of team members, and how they affect a variety of outcomes. Huber et al. offer insights into the CS and virtue profiles of medical students and physicians, a previously unexamined population of medical professionals. They subsequently explore relationships and effects on well-being and work engagement.

The final study in this section expands into a wider variety of domains, as Wagner et al. explore the associations between CS and CS-related behaviors, and excelling in the domains of work, education, relationships and leisure. They discuss differences in CS profiles across these domains, considering the interplay and effects of these strengths on a person flourishing.

## Breaking New Ground

The current collection also includes articles that take CS into previously unchartered territories, inaugurating a novel sub-field of spiritual positive psychology. Littman-Ovadia and David ignite a discussion that suggests expanding positive psychology into spirituality, touching on the paradoxes of the non-dual, framing VIA CS as the classification of the human spirit, and unleashing their potential as both pathways into and derivatives of the spiritual life. Niemiec et al. further expand the discussion of parallels between CS and spirituality, offering a complimentary discussion on the capacity of CS to promote and deepen spiritual practices and vice versa, exploring various levels and avenues of integrating spirituality in the VIA framework, including the consideration of a novel superordinate virtue.

Closely related to spiritual positive psychology are suggestions originally made regarding CS's value-laden and moral nature (Peterson and Seligman, 2004). Lavy and Benish-Wesiman propose a framework that links CS and values, suggesting CS can serve as behavioral and social manifestations. Initial empirical support is then provided, presenting the mediating role of the CS of gratitude between the value of self-transcendence and peer-rated prosocial behavior and peer acceptance in adolescent samples. Finally, Stahlmann and Ruch directly tackle the moral criterion of the VIA CS that theoretically distinguishes these traits from others, such as talents and abilities. In creating ultra-short stories describing CS-related behaviors, with and without positive consequences, they present initial evidence that suggests that all CS are rated as positively moral (albeit to different degrees) by laypersons.

## Discussion

Looking back at the current state of scientific work on CS (including the articles in this collection), we conclude that great strides have been made to consolidate CS science as a relevant sub-domain of positive psychological research, holding great potential to contribute to the cultivation of the good life. Looking forward, we encourage CS scholars to continue their line of work, addressing one or several of the following five avenues for future research.

First, an important instance in CS research includes the very criteria that define them, examples including their fulfilling nature (i.e., contributing to individual's satisfaction and happiness), and the moral value of these traits in their own right, regardless of the benefits they may entail [see Peterson and Seligman (2004), for a full review]. While certain criteria, like the former, have been robustly researched, the latter has not undergone systematic empirical examination until this collection of articles—almost two decades into CS research. Another criterion demanding greater empirical attention includes CS as elevating and non-diminishing others, and calls to address this have been made previously (Freidlin and Littman-Ovadia, 2020). Future research would benefit from revisiting the work conducted under the various criteria, identifying understudied areas, and setting the stage for bridging the gaps to gain a deeper and wider understanding of CS. Research on the classification itself should also continue to refine our knowledge of CS and develop updated versions, in the same way, the Diagnostic and Statistical Manual of Mental Disorders (DSM) classification has evolved over the years. As Peterson and Seligman (2004) state, “we anticipate that our classification of strengths will similarly evolve, by adding or deleting specific strengths of character, by combining those that prove redundant, by reformulating their organization under core virtues, and by more systematically evaluating them *vis-à-vis* our 10 criteria” (p. 31).

Second, while initial research on CS has provided valuable knowledge on the prevalence and associations of these traits with various positive outcomes, it has entered a second phase in the last decade, expanding through applied research into new areas of inquiry and advanced methodologies. It is of paramount importance that scholars pursue this path and conduct robust studies using experimental and longitudinal designs to allow for a causal explanation of purported relations (Ghielen et al., 2018; Schutte and Malouff, 2019), and provide professionals with intervention protocols on which they can confidently rely (Bakker and van Woerkom, 2018; Ruch et al., 2020).

Third, we suggest that there is great potential in further expanding our research focus by exploring the novel antecedents and outcomes of CS and virtues. In terms of antecedents, scholars may seek inspiration in the extensive work on the individual and environmental factors that influence the development of personality (Wrzus and Roberts, 2016), or talent and expertise (Gagné, 2015; Ullén et al., 2016). In terms of outcomes, we would like to emphasize ambitions previously delineated for positive psychology (Seligman, 2019) and encourage research that looks beyond the benefits of CS for individual well-being and functioning, to explore benefits for relationships, groups, communities, society, and our planet.

Fourth, we foresee a particularly important role for multi-level theorizing, -data, and -analysis in advancing the science of CS and virtues. On one hand, a multi-level lens is needed to expand our hitherto limited understanding of the composition, use, and value of individual CS in groups, such as study groups, project/work teams, or communities (see Gander et al. in this collection as an example). On the other hand, a multi-level lens is required to gain novel insights into *within-person* processes and *between-person* factors that contribute to change in CS and virtues. To date, little is known about both long-term- (i.e., development) and short-term change (i.e., moment-to-moment fluctuations) in individual CS and virtues, as well as their application.

Fifth, the COVID-19 pandemic of 2020 brought with it enormous challenges, opening up novel opportunities for the research and practice of CS. While our current understanding of CS is an understanding of the construct in times of prosperity, we hope that future research will lead to novel insights into CS in times of crises and hardships (see Martinez-Marti et al. in this collection as an example). The past year has shown that understanding the benefits of CS for personal resilience and post-traumatic growth is more relevant than ever. The benefits of adapting to change and dealing with vague and unfamiliar situations warrants an in-depth examination. CS undoubtedly plays a role in dealing with crises on the personal level, but what happens on the communal, national and international levels? What is the role of strengths in dealing with loneliness and physical distance, in individuals from different age and personal status groups? The answers to these questions are important for the development of a stronger and more cohesive community. We see an opportunity to expand the place of CS in building a better human future, while learning from past experiences.

## Conclusion

A year ago, we initiated a call for collecting articles in the field, with a desire to mark and celebrate 20 years of CS research and practice. We aimed to examine research conducted to date and lay foundations for future developments. Following the world pandemic, which began shortly after the call for the current RT, the way we live together, interact, work, educate our children, and travel changed drastically. Strengths play a key role both in a prosperous society and a society in crisis and distress, and we must gain deeper knowledge on how to utilize them as paths to creating a better, stronger, and more moral human society.

## Author Contributions

All authors listed have made a substantial, direct and intellectual contribution to the work, and approved it for publication.

## Conflict of Interest

The authors declare that the research was conducted in the absence of any commercial or financial relationships that could be construed as a potential conflict of interest.

## Footnotes

^1^VIA originally stood for “Values in Action” however the name was changed to emphasize the focus of this work which is the scientific exploration of character, not values per se. “VIA” is a word that stands on its own, in Latin meaning “the path” (Littman-Ovadia and Niemiec, 2016).
